# DNA barcoding to identify leaf preference of leafcutting bees

**DOI:** 10.1098/rsos.150623

**Published:** 2016-03-02

**Authors:** J. Scott MacIvor

**Affiliations:** Department of Biological Sciences, University of Toronto Scarborough, 1265 Military Trail, Toronto, Ontario, Canada M1C 1A5

**Keywords:** *Megachile*, Megachilidae, ITS2, rcbL, phylogeny, antimicrobial properties

## Abstract

Leafcutting bees (*Megachile*: Megachilidae) cut leaves from various trees, shrubs, wildflowers and grasses to partition and encase brood cells in hollow plant stems, decaying logs or in the ground. The identification of preferred plant species via morphological characters of the leaf fragments is challenging and direct observation of bees cutting leaves from certain plant species are difficult. As such, data are poor on leaf preference of leafcutting bees. In this study, I use DNA barcoding of the rcbL and ITS2 regions to identify and compare leaf preference of three *Megachile* bee species widespread in Toronto, Canada. Nests were opened and one leaf piece from one cell per nest of the native *M. pugnata* Say (*N*=45 leaf pieces), and the introduced *M. rotundata* Fabricius (*N*=64) and *M. centuncularis* (L.) (*N*=65) were analysed. From 174 individual DNA sequences, 54 plant species were identified. Preference by *M. rotundata* was most diverse (36 leaf species, *H*′=3.08, phylogenetic diversity (*pd*)=2.97), followed by *M. centuncularis* (23 species, *H*′=2.38, *pd*=1.51) then *M. pugnata* (18 species, *H*′=1.87, *pd*=1.22). Cluster analysis revealed significant overlap in leaf choice of *M. rotundata* and *M. centuncularis*. There was no significant preference for native leaves, and only *M. centuncularis* showed preference for leaves of woody plants over perennials. Interestingly, antimicrobial properties were present in all but six plants collected; all these were exotic plants and none were collected by the native bee, *M. pugnata*. These missing details in interpreting what bees need offers valuable information for conservation by accounting for necessary (and potentially limiting) nesting materials.

## 1. Background

The Megachilidae is the second largest bee family with over 3900 species and a worldwide distribution [1,2]. These bees are solitary and important pollinators in most terrestrial environments. Many of these bees use foreign materials to construct their nests [1–4]. For some megachilid species, these nesting materials consist of little more than fine particle mud, sand and smaller pebbles [5]. Others depend on plant materials including leaves, flower petals, resins and plant hairs [5–10]. The collection of foreign material is one factor that may have promoted the diversification of megachilid bees [4].

Megachilid bees and especially those in the genus *Megachile* predominantly collect leaves by cutting pieces with their mandibles to construct their nests [11–13]. Not all leafcutting bees actually collect leaves, some use flower petals (e.g. *M. montivaga* Cresson) [14,15] and others collect plant resins (*M. sculpturalis* Smith, *M. campanulae* Robertson), or even tile caulking and plastic bags [16]. Although leafcutting bees are well-studied compared with other solitary bees [17,18], surprisingly little information is available on which leaf types are cut and by which bee species.

Nesting material choice may limit the geographical ranges and/or abundances of particular bee species. However, the leaf preferences of megachilid bee species have rarely been investigated. Identifying leaf preference could inform management and conservation [19], research and the improvement of experimental design [20–22], as well as plant selection and horticulture to reduce damage to ornamental plant species [23]. For example, Horne [19] experimentally evaluated the leaf preference of *M. rotundata* among 11 plant species. In enclosures, bees cut all plant species offered to them but showed a significant preference for leaves of buckwheat (*Fagopyrum esculentum* Moench), which were also significantly larger than the leaves of the other 10 species. Kim [20] observed *M. apicalis* Spinola visiting *Wisteria* sp. for leaf pieces in nature and subsequently used the plant in pots in enclosures to study relationships between female body size and fecundity. Finally, Nugent & Wagner [23] compared the level of leaf defoliation by unidentified *Megachile* bee species on seven different *Populus* cultivars and found one cultivar was defoliated significantly less than all others. Mechanical properties of leaves are evidently important for leaf preference in leaf-collecting *Megachile* bees. However, studies of resin- and trichome (leaf hair)-collecting megachilid bees suggest antimicrobial factors are important to protect brood from diseases, moulds and parasites [24–26], and so these properties might also be desirable among leaf-collecting *Megachile* bees.

**Figure 1.**
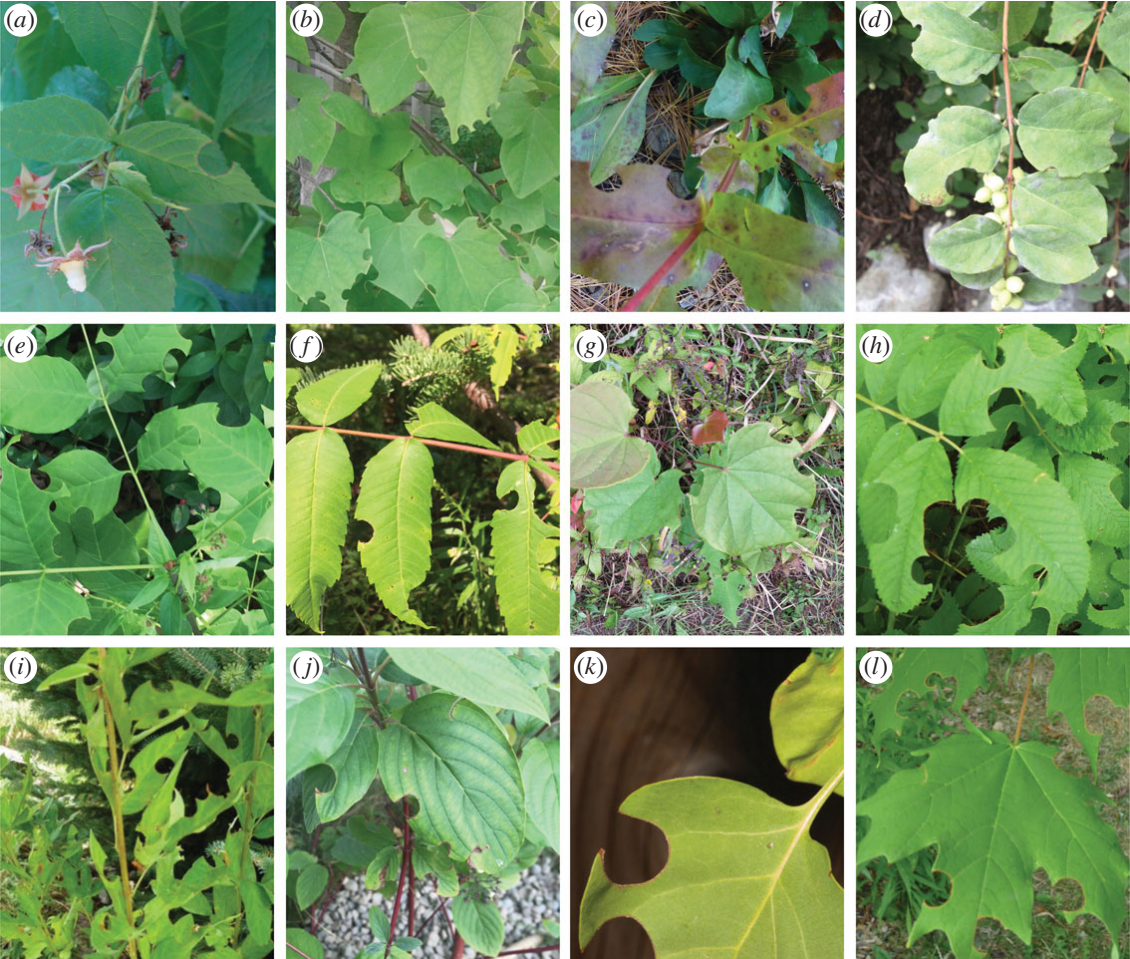
Examples of cuts by leafcutting bees on various plants. (*a*) Raspberry (*Rubus*) (photo: Sara Schraf), (*b*) redbud (*Cercis*) (photo: Heather Lynn), (*c*) beardtongue (*Penstemon*) (photo: Deb Chute), (*d*) honeysuckle (*Lonicera*), (*e*) ash (*Fraxinus*), (*f*) sumac (*Rhus typhina*), (*g*) basswood (*Tilia*), (*h*) rose (*Rosa*) (photo: Deb Chute), (*i*) Canadian tick trefoil (*Desmodium*) (photo: Deb Chute, (*j*) dogwood (*Cornus*), (*k*) lilac (*Syringa*) (photo: Rob Cruickshanks) and (*l*) maple (*Acer*) (photo: Victoria MacPhail).

Studies that report the identities of leaves cut by megachilid bees do so based on the characteristic damage to leaves [19,27–32] (figure 1). For example, one study made observations on *Hoplitis producta* (Cresson) collecting *Fragaria* sp. leaf pieces [33], Michener [1] noted *Hoplitis pilosifrons* (Cresson) using *Oenothera* sp. leaves, *M. centuncularis* was observed cutting leaves from beech (*Fagus*) trees [34], and another study found the ground-nesting *M. integra* Cresson using blackberry (*Rubus* sp.) [35]. Strickler *et al.* [32] linked *M. relativa* Cresson to cutting plants in the genera *Fragaria* and *Epilobium*, and *M. inermis* Provancher to (among many others) *Acer*, *Betula*, *Rubus* and *Amelanchier*. The authors associated the size of the cuts on the leaves to the two different bees studied because the first bee species cut noticeably larger pieces than the second one.

Determining leaf preference from observations of the bee in the act of cutting, or by surveying for the characteristic leaf cuts on plants, gives partial lists that can offer useful details about local megachilid bee nesting requirements. However, methods that more accurately link leaf–bee species interactions could provide details about inter- and intraspecific variation in leaf preference, contributing to gaps in knowledge on these essential pollinators. One technique that could ameliorate our understanding of leafcutting bee nesting preferences is DNA barcoding, which uses the diversity in short gene sequence regions to improve species-level identification [36–39]. These techniques are especially useful in cases where morphological identification is difficult or not possible [40], as is the case with leaf fragments in bee nests.

In this study, I use DNA barcoding to identify leaf preference of three leafcutting bee species, one native (*M. pugnata* Say) and two exotic bees that are naturalized in the study region, *M. centuncularis* (L.) and *M. rotundata* [13]. The leaf fragments were removed from nests constructed in artificial nest-boxes that were set up in the city of Toronto in 2012 and 2013. I first compare the species diversity in leaf preference among the three bee species and as previous studies show bee species tend to prefer leaves from only a few species [19,20], I hypothesize that bees would not overlap in their leaf preferences. Second, because anthropogenic landscape change can reduce phylogenetic diversity (pd) [41], I hypothesize that the leaf preference of the native bee studied in an urban environment would be more phylogenetically constrained than that of the two exotic bees. Native bees prefer native plants [42,43], and so similarly I hypothesize that the native bee (*M. pugnata*) would prefer native leaves rather than exotic ones for nesting material. Lastly, I determine whether any bees exhibit specialization or affinities for native or exotic species, or particular kinds of vegetation, including trees, shrubs or flowering plants, which would have applications for conservation planning and management.

## 2. Material and methods

### 2.1 Sample collection

To sample leaves from nests I set up artificial nests (nest-boxes), which are a preferred habitat for many cavity-nesting bee species [8,17,44,45]. One nest-box was set up at each of 200 locations throughout the city of Toronto in residential gardens, community gardens, public parks and on green roofs, each year from 2011–2013, approximately 250 m apart in distance [46]. Each nest-box was made of a 30 cm piece of PVC pipe that was 10 cm in diameter into which 30 cardboard nesting tubes (10 of each of three widths: 7.6 mm, 5.5 mm, 3.4 mm) were inserted [47]. These were attached to fixed structures at each site (e.g. wooden stake, fence post, exposed tree limb). Nest-boxes were set up in April and retrieved in October. The nesting tubes were opened and all brood were stored in a walk-in fridge at 4^°^C for the winter. In spring, all individual brood cells were placed in a growth chamber at 26^°^C and 65% humidity so that they could be identified to species level after emergence.

**Figure 2.**
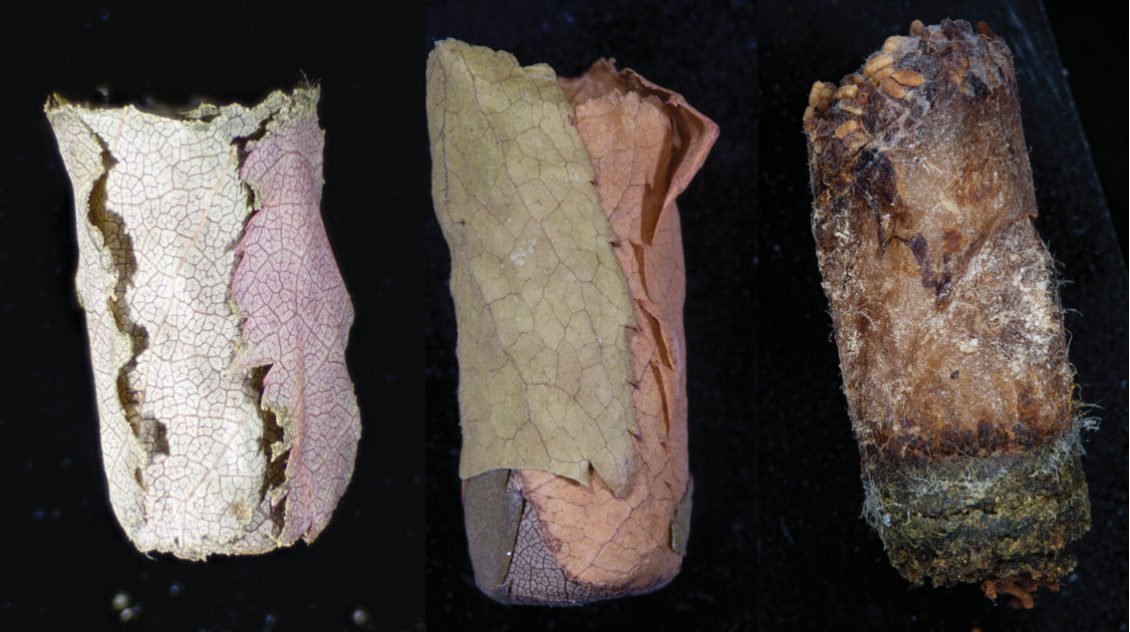
Representative examples of brood cells constructed by each of the leafcutting bees. From left to right: *M. rotundata*, *M. centuncularis* and *M. pugnata*. Photos were taken at the Packer Collection at York University (PCYU) laboratory using a Canon E05 40D camera with a K2 lens and a 10× lens attachment, with a Microoptics ML 1000 fibreoptics illuminating system at the highest flash setting and highest magnification.

Thirty-six bee species (including five cleptoparasites) used the nest-boxes over the three seasons [46]. From this group, three common leafcutting species were selected for study, the native *M. pugnata*, and the introduced *M. centuncularis* and *M. rotundata*. *M. pugnata* uses mud and chewed leaves to line its brood cells, and makes partitions between adjacent cells using circular pieces of leaves laid one over the other (figure 2). *M. rotundata* and *M. centuncularlis* collect circular pieces of leaves and line each brood cell with a roll of layered leaves (figure 2). One leaf piece was selected from one cell per nest of the native *M. pugnata* (*N*=45 leaf pieces; 10 sites), and the introduced *M. rotundata* (*N*=64; 29 sites) and *M. centuncularis* (*N*=65; 31 sites). A total of 62 sites were sampled over the 2012 and 2013 seasons. No sites were shared among all three bees, and only two sites were shared between *M. pugnata* and *M. centuncularis*, one between *M. pugnata* and *M. rotundata*, and four sites between *M. centuncularis* and *M. rotundata*.

To determine the identity of each leaf cut sample, using forceps I removed one leaf piece per brood cell, then each was cut into a 0.5 cm^2^ square, washed in ethanol, air dried, and placed into an individual sampling vial in a 96-vial DNA extraction plate (AcroPrep) provided by the Canadian Centre for DNA Barcoding (CCDB). Two filled plates of leaf fragments were then sent to the CCDB for DNA extraction [48] and barcoding [37,49,50]. The primers rcbL and ITS2 were used to obtain nucleotide sequences for each sample. Identities of the leaf samples were determined by downloading sequence data (greater than 50 bp) from the Barcode of Life Data (BOLD) systems. A total of 47.4% of samples yielded incomplete sequence data (rcbL=11.9% of sample, ITS2=35.6%). However, sequence quality was high (rbcL=96.7% (<1% Ns); ITS2=93.75% (<1% Ns)) and sequence data were cross-referenced in the gene sequence database GenBank [51] for 100% matches using the Basic Local Alignment Search Tool (BLAST) [52]. Lastly, once the identities of the leaves were determined, the antimicrobial properties of each of the plant species were determined by surveying the literature that measures these values (table 1) [53–60].

**Table 1.** List of leaf species collected by each of the three megachilid bees as determined using DNA barcoding. Plants having secondary compounds with known antimicrobial properties were determined for each species or genus as per ^a^Nickell [53], ^b^Borchardt *et al*. [54], ^c^Mogg *et al*. [55], ^d^Cappuccino & Arnason [56], ^e^Hayes [57], ^f^Madsen & Pates [58], ^g^Amadou *et al*. [59], ^h^Chen *et al*. [60]. These were denoted at the species level with ‘++’, at the genus level with ‘+’. Species known to have no known antimicrobial and antifungal properties are given ‘−’, and at the genus level ‘−−’. MC, *M. centuncularis*; MP, *M. pugnata*; MR, *M. rotundata*.

family	genus	common	status	type	antimicrobial	MC	MP	MR
Anacardiaceae	*Rhus typhina* L.	staghorn sumac	native	tree	++^ab^	1	0	4
Apocynaceae	*Cynanchum rossicum* (Kleopow) Borhidi	dog-strangling vine	exotic	perennial	++^cd^	0	0	7
Asteraceae	*Artemisia biennis* L.	wormwood	exotic	biennial	++^a^	0	0	1
	*Helianthus annuus* L.	common sunflower	native	annual	+^a^	0	0	1
	*Rudbeckia triloba* L.	brown-eyed susan	native	perennial	+^a^	0	0	1
	*Solidago canadensis*L.	Canadian goldenrod	native	perennial	++^a^	0	0	1
	*Symphyotrichum nova-angliae*(L.) G.L.Nesom	New England aster	native	perennial	++^a^	0	1	0
Caryophyllaceae	*Silene vulgaris*(Moench) Garcke	bladder campion	exotic	perennial	−−^b^	1	0	0
Celastraceae	*Celastrus scandens* L.	American bittersweet	native	tree	++^a^	1	0	0
Chenopodiaceae	*Chenopodium album* L.	lamb’s quarter	exotic	annual	++^a^	0	2	1
Convolvulaceae	*Convolvulus arvensis* L.	field bindweed	exotic	annual	++^e^	0	0	1
	*Ipomoea hederacea*Jacq.	ivy-leaved morning glory	exotic	annual	+^f^	0	0	2
Cornaceae	*Cornus stolonifera*L.	red osier dogwood	native	shrub	++^a^	0	1	1
	*Cornus racemosa* Lam.	gray dogwood	native	shrub	+^b^	0	5	0
Euphorbiaceae	*Euphorbia dentata* Michx.	toothed spurge	exotic	annual	+^b^	0	0	1
Fabaceae	*Amphicarpaea bracteata* (L.) Fernald.	hog peanut	native	perennial	++^a^	0	0	1
	*Baptisia australis* Hort. Ex. Lehm.	false indigo	exotic	perennial	++^a^	1	0	1
	*Caragana arborescens*Lam.	Siberian peashrub	exotic	shrub	+^a^	1	0	0
	*Cercis canadensis* L.	redbud	native	tree	++^a^	5	0	3
	*Desmodium canadense*(L.) DC.	Canadian tick trefoil	native	perennial	++^a^	2	0	2
	*Lotus corniculatus*L.	bird’s-foot trefoil	exotic	perennial	−−^b^	0	0	2
	*Melilotus alba*Medik.	sweet clover	exotic	annual	++^a^	0	0	4
	*Phaseolus vulgaris* L.	string bean	exotic	annual	++^a^	4	0	0
	*Robinia pseudoacacia*L.	black locust	native	tree	++^a^	2	0	0
	*Securigera varia* (L.) Lassen	crown vetch	exotic	annual	++^a^	0	0	1
Grossulariaceae	*Ribes hirtellum*Michx.	American gooseberry	native	shrub	++^a^	0	0	1
Juglandaceae	*Carya cordiformis* (Wangenh.) K. Koch	bitternut hickory	native	tree	+^a^	0	1	0
Lamiaceae	*Stachys palustris* L.	marsh woundwort	exotic	perennial	−−^b^	1	0	1
Lythraceae	*Lythrum salicaria* L.	purple loosestrife	exotic	perennial	++^b^	1	5	0
Oleaceae	*Syringa vulgaris* L.	common lilac	exotic	tree	−−^b^	0	0	3
Onagraceae	*Epilobium ciliatum* Raf.	slender willowherb	native	perennial	++^b^	0	1	0
	*Epilobium parviflorum*(Schreb.) Schreb.	smallflower hairy willowherb	native	perennial	+^b^	2	0	1
	*Oenothera biennis*L.	common evening primrose	native	biennial	++^be^	0	0	2
	*Oenothera parviflora*L.	northern evening primrose	native	biennial	++^a^	1	0	0
	*Oenothera pilosella* Raf.	meadow evening primrose	native	perennial	+^b^	0	5	1
Papaveraceae	*Chelidonium majus* L.	greater celandine	native	perennial	++^a^	0	0	2
Poaceae	*Setaria italica* (L.) P. Beauv.	foxtail millet	exotic	annual	++^g^	0	0	1
Polygonaceae	*Fallopia convolvulus* (L.) Á. Löve	black bindweed	exotic	annual	++^h^	0	1	1
Rhamnaceae	*Rhamnus cathartica* L.	common buckthorn	exotic	tree	−−^b^	0	0	2
Rosaceae	*Crataegus macrosperma*Ashe	big fruit hawthorn	native	tree	+^b^	0	1	0
	*Fragaria virginiana*Duchesne	wild strawberry	native	perennial	+^b^	0	1	0
	*Prunus avium* (L.)	bird cherry	exotic	tree	+^b^	2	4	1
	*Prunus virginiana*L.	chokecherry	native	tree	+^b^	0	1	0
	*Rosa blanda*Gray	smooth wild rose	native	shrub	+^b^	1	1	1
	*Rosa palustris*Marshall	swamp rose	exotic	shrub	+^b^	3	4	0
	*Rosa multiflora*Thunb.	multiflora rose	native	shrub	++^a^	15	5	5
	*Rosa glauca* Pourret	red-leaved rose	exotic	shrub	+^b^	3	1	1
	*Rosa virginiana*P.Mill	common wild rose	exotic	shrub	+^b^	0	5	0
	*Rubus occidentalis* L.	black raspberry	native	shrub	+^b^	3	0	0
Sapindaceae	*Acer saccharum*Marshall	sugar maple	native	tree	++^a^	4	0	3
	*Acer platanoides* L.	Norway maple	exotic	tree	++^a^	8	0	1
Tiliaceae	*Tilia cordata*Mill.	small leaf linden	native	tree	+^b^	0	0	1
Vitaceae	*Ampelopsis japonica* (Thunb.) Makino.	Japanese peppervine	exotic	shrub	−^g^	1	0	0
	*Vitis riparia* Michx.	riverbank grape	native	shrub	+^ad^	2	0	1

### 2.2 Analysis

From the type and number of leaf species cut as determined by DNA barcoding, I determined the richness and Shannon diversity index (*H*′) of leaf species preference for each of the three bee species. Rarefaction curves for leaf preference of all bee species were interpreted using iNEXT software [61,62]. Using the package ‘ecodist’ [63] in the R statistical program [64], I calculated the Bray–Curtis dissimilarity index to compare how leaf preference differed between the leafcutting bee species. I then used a Spearman’s rank correlation to determine similarity in leaf preference between each bee species pair. Bee species pairs that were positively correlated meant they overlapped in leaf preference. The leaf preferences of the three bee species were also compared by plant species grouped by status (‘exotic’ or ‘native’) and type (‘tree’, ‘shrub’, ‘perennial’, ‘annual’) using a set of Pearson’s *χ*^2^-tests in the R package ‘MASS’. Some plant species used by the bees were biennials (*N*=3, see table 1) and these were included as perennials in the analysis. Another *χ*^2^-test was used to compare preference with plant species grouped as ‘woody’ (‘tree’ + ‘shrub’) or ‘non-woody’ (‘perennial’ + ‘biennial’ + ‘annual’).

To compare pd in leaf preference by each of the three bee species, I constructed a phylogeny of all leaf species identified from the nests of each species by using the prune function in the R package ‘phytools’ [65] from an existing rooted phylogeny of 912 plant species identified from the study region [66]. Faith’s pd was calculated from the phylogeny of leaf preference for each of the three bee species using the R package ‘picante’ [67].

## 3. Results

**Figure 3.**
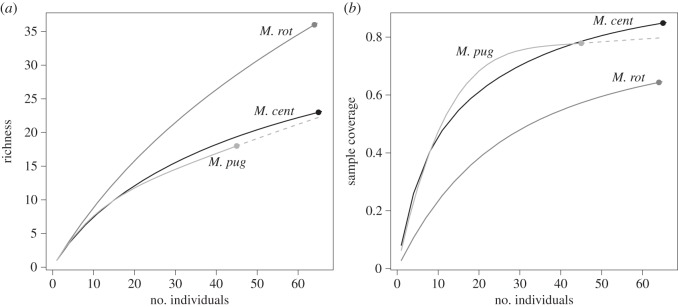
(*a*,*b*) Rarefaction and species completeness curves for the leafcutting bee species (*M. centuncularis*=*M. cent*, *M. pugnata*=*M. pug*, *M. rotundata*=*M. rot*).

DNA barcoding identified leaves of 54 species in 46 genera and 24 families (table 1) from 174 samples (e.g. every third sample was of a unique plant species). The leaves collected were significantly different among bee species (*χ*^2^=197.44, *p*<0.001). *M. centuncularis* collected 23 species in 20 genera and 10 families, and 23.1% of the total leaves analysed were *Rosa multiflora* Thunb. *M. pugnata* collected leaves from 18 species in 14 genera and 8 families, with *Cornus racemosa* Lam., *Lythrum salicaria* L., *R. multiflora* and *R. virginiana* P.Mill. all tied for the most identified leaf type (11.1% each). *M. rotundata* collected leaves from 36 species in 33 genera and 20 families. *Cynanchum rossicum* (Kleopow) Borhidi was the most often identified species for *M. rotundata*. Rarifying leaf species richness and sample completeness for all three bees to control for uneven sample size indicated that the estimate of total species richness was lower for *M. pugnata* and *M. centuncularis* than *M. rotundata* and that sampling more adequately characterized the leaf preference of *M. pugnata* than that of *M. centuncularis* and *M. rotundata* (figure 3).

**Figure 4.**
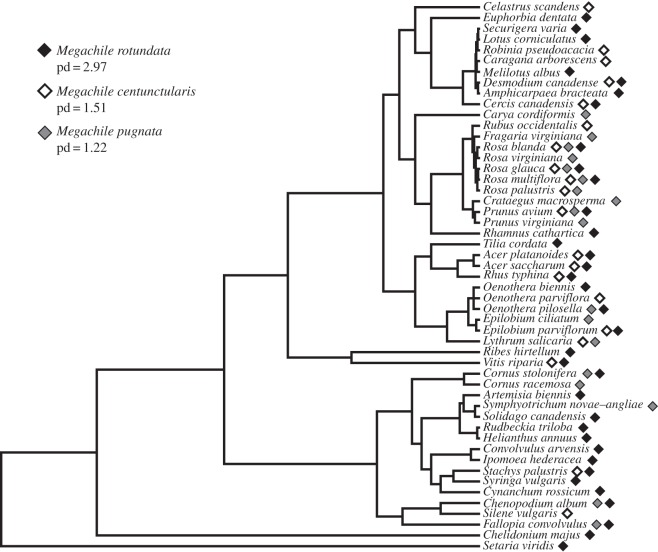
Phylogeny of leaf preferences by the leafcutting bee species. The diamonds indicate by which bee species the respective leaf species are selected.

Diversity in leaf preference was greatest in the exotic *M. rotundata* (*H*′=3.08) then *M. centuncularis* (*H*′=2.38), and lowest in the native *M. pugnata* (*H*′=1.87). The pd in leaf preference was highest in *M. rotundata* (*pd*=2.97), followed by *M. centuncularis* (*pd*=1.51) and the native *M. pugnata* (*pd*=1.22) (figure 4). Controlling for species richness (pd/SR), *M. centuncularis* (0.64) and *M. pugnata* (0.65) were more constrained in pd than was *M. rotundata* (0.96).

**Figure 5.**
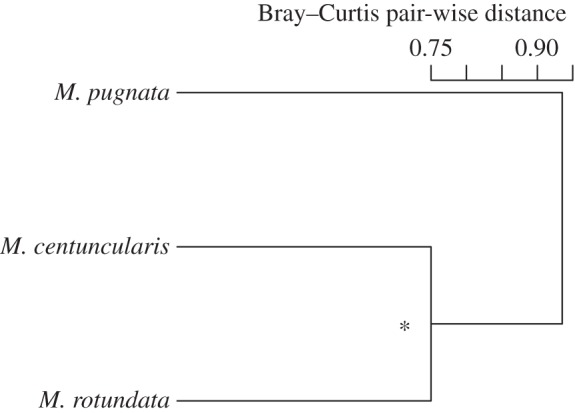
Bray–Curtis cluster tree showing the similarity in leaf preference between the three bee species.

A Bray–Curtis cluster analysis showed leaf preference between *M. centuncularis* and *M. rotundata* was more similar than that of *M. pugnata* (figure 5). A Pearson’s *R* correlation confirmed that there was significant overlap in the leaf preference of *M. centuncularis* and *M. rotundata* (*R*=0.288, *p* = 0.035). Four plant species were visited by all bee species and all were shrubs or trees: *R. blanda* Gray, *R. glauca* Pourret, *R. multiflora* and *Prunus avium* (L.) (table 1). Twelve plant species were shared between *M. centuncularis* and *M. rotundata*, whereas *M. pugnata* shared six and eight leaf species with *M. centuncularis* and *M. rotundata*, respectively. *M. rotundata* used seven species from the legume family Fabaceae and preferred these plants to the others (21.5% of the total). Rosaceae was the most used family for both *M. centuncularis* (6 species, *N*=27) and *M. pugnata* (9 species, *N*=23). For *M. pugnata*, leaves in the family Rosaceae represented half of all species collected. Leaves of *R. multiflora* were the most abundantly collected species by all three species combined (*N*=25). Only one grass species was collected, *Setaria italica* (L.) P. Beauvois, by one *M. rotundata* individual (table 1).

Native leaves were not collected more than exotic leaves (*χ*^2^=1.30, *p*=0.52) with 30 native plant species and 24 exotic species collected among the three bees (table 2). Exotic plant species collected included cosmopolitan plants such as lamb’s quarter (*Chenopodium album* L.), bird’s foot trefoil (*Lotus corniculatus* L.) and crown vetch (*Securigera varia* (L.) Lassen) as well as plants invasive to the Southern Ontario region, including purple loosestrife (*L. salicaria* L.) and dog-strangling vine (*C. rossicum*) (table 1). Interestingly, all native plants collected by the bees are known to have antimicrobial properties (table 1). Only six plant species collected were known to contain no antimicrobial properties: *Silene vulgaris* (Moench) Garcke, *L. corniculatus* L., *Stachys palustris* L., *Syringa vulgaris* L., *Rhamnus cathartica* L. [54] and *Ampelopsis japonica* (Thunb.) Makino [59]. All six of these plants were exotic to the study region, and none were used by the native bee, *M. pugnata* (table 1). Lastly, there was a significant difference among bees’ preference for different plant types (‘tree’, ‘shrub’, ‘perennial’, ‘annual’) (*χ*^2^=29.61, *p*<0.001; table 2), with *M. centuncularis*, in particular, visiting ‘woody’ plants (*N*=49) significantly more than ‘non-woody’ plants (*N*=13) (*χ*^2^=19.24, *p*<0.001; table 2).

**Table 2.** The status (native or exotic) and types of vegetation collected by the three leafcutting bee species. The percentage of the total number of plants identified and their abundances (in parentheses) are given. An *indicates a significant difference in the leaf preference of *M. centuncularis* between woody and non-woody plant species.

	*M. pugnata*	*M. centuncularis*	*M. rotundata*
native	48.9% (22)	38.5% (25)	53.1% (30)
exotic	51.1% (23)	61.5% (40)	46.9% (34)
woody	64.4% (29)	75.4% (49)*	42.2% (27)
tree	15.6% (7)	35.4% (23)	23.4% (15)
shrub	48.8% (22)	40.0% (26)	18.8% (12)
non-woody	35.6% (16)	24.6% (16)	57.8% (37)
perennial	28.9% (13)	18.5% (12)	37.5% (24)
annual	6.7% (3)	6.1% (4)	20.3% (13)

## 4. Discussion

Leafcutting bees are reported to be selective in leaf preference, and to forage on only a few plant species [19,20]. Past studies reporting leaf preference of leafcutting bees did so using observational data [15,32,34]. In this study, I use DNA barcoding to identify leaf pieces from nests of three leafcutting bees and demonstrate that diversity in the leaves selected is far greater than previously reported. The leaf preferences of the two exotic bees overlap significantly and so I reject my first hypothesis that leaf preferences would be different among bee species. The pd and the species spectrum of selected leaves were higher in the two exotic bee species than in the native one. This is in accordance with the second hypothesis that leaf preference of the native bee is more restricted to phylogenetic groups. Finally, according to the *χ*^2^-tests none of the bee species exhibited a significant preference for native or exotic leaf species, indicating that the third hypothesis, e.g. that the native bee, *M. pugnata*, would prefer native plant species to exotic species, is not valid.

Dependence of the native *M. pugnata* on nesting materials that are more phylogenetically related could make this species and other native bees more susceptible to environmental change [68]. Ecologists increasingly use phylogenetic measures to inform our understanding of community assembly as well as management practices to conserve biodiversity and ecological functioning [69,70]. Some studies have used phylogenetic relatedness to describe bees and their foraging preferences [71,72] and their nesting behaviour [73]. Future work could evaluate matching (or mis-matching) in phylogenetic relationships (e.g. [74]) among bees and nesting resources. These relationships examined along gradients of environmental change could aid in determining the extent to which these eco-evolutionary relationships depend on environmental filters, such as urbanization [75].

The leaves collected belonged to a variety of plant types: trees, shrubs and flowering plants, as well as one grass species. Only *M. centuncularis* exhibited a significant preference for woody plants (e.g. trees and shrubs) (table 2). None of the bee species exhibited significant preference for native or exotic leaf types. *M. pugnata* visited many exotic species including spontaneous urban plants (e.g. lamb’s quarter (*Chenopodium album*)) and invasive species (e.g. purple loosestrife (*L. salicaria*)) (table 1). The exotic *M. rotundata* collected more exotic leaf types than the other two species (table 2), and among the 36 species recorded was the invasive dog-strangling vine (*Cyananchum rossicum*) [76] (table 1). This vine has invaded Southern Ontario, where it ‘strangles’ and suppresses the growth of native vegetation [77,78]. A number of other exotic plants were visited for nesting material by the three bee species ((e.g. bird’s foot trefoil (*L. corniculatus* L.) and sweet clover (*Melilotus albus* Medik.)) (table 1). As exotic flora is abundant in urban areas [79,80], urban landscapes could provide a wider range of nest material for leafcutting bees, including native species [81,82].

Bee diversity and abundance are strongly linked to characteristics of the local environment, including the presence and quantity of foraging and nesting materials, as well as the amount of, and distance between, habitat containing these resources [83–87]. In urban landscapes containing many thousands of individually managed gardens that together form a rich diversity of flowering plants [88,89], leafcutting bees are not limited in these areas by nesting materials. Given how diverse leaf selection was for each of the leafcutting bees in this study, it is possible that further DNA barcoding would identify even greater numbers of plant species used. Citizen science to help record leaf cuts on plants (see figure 1) with DNA barcoding could be useful for examining leaf preference and diversity along urban–rural gradients where dominant factors affecting plant species assembly change from natural to anthropogenic [75,90].

Almost all the leaves identified in the nests are known to contain antimicrobial properties (table 1). Many ground-nesting bees waterproof and sterilize their brood cells using glandular secretions from the Dufour’s glands [91,92]. These glands are significantly reduced in above-ground-nesting bees including megachilids [2], and so antimicrobial properties of leaves might inform choice [24–26]. One above-ground-nesting megachilid, *Anthidium manicatum*, collects leaf trichomes to make their nests which have antimicrobial properties [25] and their physical properties actively prevent attacking parasites [93]. One study showed leaf type and physical properties impact choice in *M. rotundata*, which preferred buckwheat unanimously over alfalfa and especially leaves greater than 1 cm^2^ [19]. A combination of the antimicrobial and mechanical properties of leaves may inform choice among leaf types and why certain leaves are preferred over others by megachilid bees.

Missing from this study is an examination of the continuity and variability in leaf choice within single nests, which limits interpretation of the diversity of leaf types used by individual females. Identifying all leaves comprising a complete nest of individual bees (some complete nests contain greater than 100 individual leaf pieces) would contribute to knowledge on how diverse leaf preference is among individual bees, or even how it changes over a season. Knowing the identities of plants used by leafcutting bees can inform ‘complete’ pollinator gardening and broader actions supporting wild pollinators that include both foraging and nesting requirements [42,94–97].

## Data accessibility

DNA sequences: http://dx.doi.org/10.5883/DS-8669.
